# The corrected gene proximity map for analyzing the 3D genome organization using Hi-C data

**DOI:** 10.1186/s12859-020-03545-y

**Published:** 2020-05-29

**Authors:** Cheng Ye, Alberto Paccanaro, Mark Gerstein, Koon-Kiu Yan

**Affiliations:** 1 Department of Computer Science, Centre for Systems and Synthetic Biology, Royal Holloway, University of London, Egham, TW20 0EX UK; 2 School of Applied Mathematics, Fundação Getulio Vargas, Rio de Janeiro, Brazil; 3 Program in Computational Biology and Bioinformatics, Department of Molecular Biophysics and Biochemistry, Department of Computer Science, Department of Statistics and Data Science, Yale University, New Haven, CT 06520 USA; 4 Department of Computational Biology, St. Jude Children’s Research Hospital, Memphis, TN 38105-3678 USA

**Keywords:** 3D genome, Hi-C data analysis, Network modularity, Network theory

## Abstract

**Background:**

Genome-wide ligation-based assays such as Hi-C provide us with an unprecedented opportunity to investigate the spatial organization of the genome. Results of a typical Hi-C experiment are often summarized in a chromosomal contact map, a matrix whose elements reflect the co-location frequencies of genomic loci. To elucidate the complex structural and functional interactions between those genomic loci, networks offer a natural and powerful framework.

**Results:**

We propose a novel graph-theoretical framework, the Corrected Gene Proximity (CGP) map to study the effect of the 3D spatial organization of genes in transcriptional regulation. The starting point of the CGP map is a weighted network, the gene proximity map, whose weights are based on the contact frequencies between genes extracted from genome-wide Hi-C data. We derive a null model for the network based on the signal contributed by the 1D genomic distance and use it to “correct” the gene proximity for cell type 3D specific arrangements. The CGP map, therefore, provides a network framework for the 3D structure of the genome on a global scale. On human cell lines, we show that the CGP map can detect and quantify gene co-regulation and co-localization more effectively than the map obtained by raw contact frequencies. Analyzing the expression pattern of metabolic pathways of two hematopoietic cell lines, we find that the relative positioning of the genes, as captured and quantified by the CGP, is highly correlated with their expression change. We further show that the CGP map can be used to form an inter-chromosomal proximity map that allows large-scale abnormalities, such as chromosomal translocations, to be identified.

**Conclusions:**

The Corrected Gene Proximity map is a map of the 3D structure of the genome on a global scale. It allows the simultaneous analysis of intra- and inter- chromosomal interactions and of gene co-regulation and co-localization more effectively than the map obtained by raw contact frequencies, thus revealing hidden associations between global spatial positioning and gene expression. The flexible graph-based formalism of the CGP map can be easily generalized to study any existing Hi-C datasets.

## Introduction

Most cell types in the human body have an identical one-dimensional (1D) genome, (i.e. a linear sequence of nucleotides), yet their genomes have different underlying 3D architectures [1]. Their different ways of packing DNA molecules into cell nuclei, in particular via the formation of loops or domains [2, 3] in association with the nuclear lamina and nuclear organelles [4, 5], lead to different spatial configurations of genomic elements in 3D. The spatial proximity between genomic elements plays a central role in gene regulation and cell fate determination [6–8], and its disruption can lead to dysregulation and cause diseases including cancer [9, 10]. Over the last decade, genome-wide ligation-based assays, such as Hi-C, have provided an unprecedented opportunity to investigate the 3D organization of the genome, and thus the spatial proximity between any genomic elements of interest [11–13]. Results of a typical Hi-C experiment are summarized in a chromosomal contact map [11]. By binning the genome into equally sized bins, the contact map is a matrix whose elements reflect the population-averaged co-location frequencies of genomic loci originated from the bins. The contact frequency can be viewed as a measurement for the probability of a Hi-C ligation between genomic loci, which could be used as a proxy for spatial proximity: the greater the frequency, the smaller the distance in space.

A natural representation for a genome-wide contact map is a graph or network. Indeed, networks have been used to represent the global structure of 3D genomes [14, 15]. Nevertheless, it is not entirely clear what is the best null model for those weighted graphs. Analysis of Hi-C, and indeed related 3C based technologies, have realized that the contact frequency observed between a pair of genes in the contact map is a mixture of two components [11, 16]. The first component, the 1D component, is related to their genomic distance, i.e., the distance between the genes due to the fact that they are positioned sequentially on the 1D DNA strand. More specifically, two genes that are next to each other on the 1D DNA strand are expected to have a higher contact frequency as compared with two genes that are farther apart. The second component, the 3D component, depends on cell specific arrangements of genes in 3D. While many Hi-C analyses have used the idea of separating these components [17, 18], this separation has been implemented at a local level, meaning that each individual pair of loci is analyzed independently of the other ones, mainly in the context of enhancer-target predictions [19]. As most human cells have effectively the same 1D genome sequence, it is the 3D component that provides a cell type specific role in gene regulation. Our idea is to derive an appropriate null model for the 1D component and to provide a network framework for the 3D structure of the genome on a global scale.

In this paper, we present a novel mathematical framework that effectively extracts the 3D component of the gene contact frequency for an entire genome and embeds it into a graph that we have called the *“Corrected Gene Proximity (CGP)”* map. Similar to some existing approaches, our procedure can be thought of as a “signal separation” procedure that is able to extract the 3D component from the mixture of 1D and 3D frequency components that constitutes the experimental Hi-C data. But, unlike previous analyses that focus on addressing the statistical significance of pairs of loci – for instance, to determine whether pairs of loci are candidates for enhancer-promoter contacts – the CGP map serves as a map of the 3D structure of the genome on a global scale. With such a global framework, we are able to analyze intra- and inter- chromosomal interactions together and perform analysis that aims to understand the global structure of the genome. Our results show that the CGP map can detect and quantify gene co-regulation and co-localization more effectively than the map obtained by raw contact frequencies and reveal hidden associations between global spatial positioning and gene expression.

## Results

### The corrected gene proximity map

Starting from a genome-wide Hi-C contact map, we extracted the contact frequencies between all protein-coding genes, forming a square matrix *W* (see Methods for details). This matrix can be viewed as the adjacency matrix of a network in which nodes represent genes and the weight of each edge corresponds to the contact frequency between the two genes it connects. We called this weighted network the *“gene proximity map”* since its weights represent spatial proximity as detected by Hi-C: the higher the weight, the smaller the distance between two genes.

As we described earlier, the weights in the network can be thought of as a combination of the 1D and 3D components of the gene contact frequency. To extract the 3D component, we began by developing a null model for the 1D component, i.e., a model for the spatial positioning of genes based exclusively on their distance on the 1D genome. We shall use *E* to denote the weight matrix for this null model. Specifically, we assume that the (*i*, *j*)-th element *E*_*ij*_, the expected number of contacts between genes *i* and *j*, takes the form:

1 $$ {\displaystyle \begin{array}{l}{E}_{ij}={k}_i{k}_jf\left({d}_{ij}\right)\\ {}\mathrm{where}f\left({d}_{ij}\right)\propto \Big\{\begin{array}{c}{f}^{\mathrm{intra}}\kern0ex (d),\mathrm{if}\ \mathrm{gene}\ \mathrm{i}\ \mathrm{and}\ \mathrm{j}\ \mathrm{are}\ \mathrm{located}\ \mathrm{at}\ \mathrm{the}\ \mathrm{same}\ \mathrm{chromosome}\ \mathrm{and}\ \mathrm{separated}\ \mathrm{by}\ \mathrm{genomic}\ \mathrm{distance}\ \mathrm{d}\\ {}{f}^{\mathrm{inter}},\kern1.36em \mathrm{i}\mathrm{f}\kern0.34em \mathrm{gene}\kern0.28em \mathrm{i}\kern0.28em \mathrm{and}\kern0.28em \mathrm{j}\kern0.28em \mathrm{are}\kern0.28em \mathrm{located}\kern0.28em \mathrm{at}\kern0.28em \mathrm{d}\mathrm{i}\mathrm{f}\mathrm{f}\mathrm{erent}\kern0.34em \mathrm{chromosome}\end{array}\operatorname{}\end{array}}. $$

There are two cases here: both gene *i* and gene *j* locate on the same chromosome; or they are on different chromosomes. For the first case, *f*^*intra*^(*d*) is a cell type specific function, numerically estimated from the genome-wide Hi-C contact map, that maps the genomic distance *d* between a pair of genes to an average contact frequency over all pairs of genomic loci that are separated by *d*. On the other hand, for any pairs of genes that belong to two different chromosomes, we assume they have equal chances to interact. So *f*^*inter*^ is a constant that is computed from the average contact frequency of all inter-chromosomal interactions (see Methods for details). We further impose the following set of constraints:

2 $$ {\sum}_j{E}_{ij}={\sum}_j{W}_{ij},\forall i. $$

These constraints ensure that the total number of contacts mapped to gene *i* empirically is the same as the number of contacts assigned to it by the null model. By imposing these constraints, *k*_*i*_ can be solved using an iterative scheme (see Methods for details). Intuitively, *k*_*i*_ can be viewed as the “visibility” of gene *i*, which is a biological parameter related to gene size, chromatin accessibility, gene location in the nucleus, etc. Genes that are long and located in the center of the nucleus will normally have higher visibility than short genes and genes located on the periphery of the nucleus. Overall, Equation (1) implies that the expected number of contacts between two genes depends on their genomic distance as well as the product of their intrinsic visibility. Highly visible genes are therefore more likely to be in contact with others.

Having defined the null model, which accounts for the 1D component of the contact frequency, we can define the CGP map, denoted by *B*, as the difference between the observed gene proximity matrix and the developed null model:

$$ B=W-E. $$

**Fig. 1:**
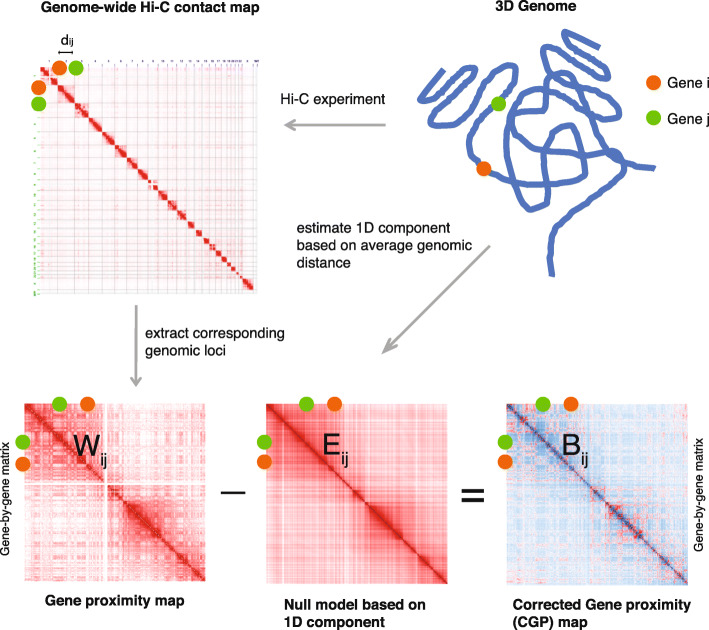
Schematic for the construction of the CGP map. Given a 3D human genome organization in which the brown and green circles represent two protein-coding genes *i* and *j* respectively, the corresponding genome-wide Hi-C contact map quantifies the contact frequencies between all possible pairs of genomic loci. Using the correspondence between genes and genomic loci, the gene proximity matrix *W* is obtained by extracting the contact frequencies between all pairs of genes. A null model, denoted as *E*, is derived from the 3D genome as well as the gene proximity map, representing the estimated gene proximity hidden in the gene contact frequencies, which is based exclusively on the positions of genes on the 1D DNA strand, i.e., the genomic distance. The CGP map, denoted as *B*, is extracted from the gene proximity map *W* by subtracting the null model *E*. Matrix element *B*_*ij*_ captures the corrected spatial proximity between genes *i* and *j*

In other words, the CGP map quantifies the corrected spatial proximity between genes by eliminating the gene contact frequency component due to the 1D genomic distance from the results of Hi-C experiments. This is motivated by the conventional definition of modularity matrix in network theory, which is defined as the difference between the adjacency matrix and a matrix explaining the expected connectivity pattern of the network, and is shown to be powerful for detecting network communities [20]. Importantly, in our analysis, *W* is the adjacency matrix of the gene proximity network and *B* can be thought of as a generalized modularity matrix for the network (see Supplementary Materials for details). Figure 1 shows a schematic of the construction of the CGP map. In our analysis, all genome-wide Hi-C contact maps had been corrected by the ICE technique [21]. Importantly, our framework can be applied to Hi-C maps corrected by any other software tools such as HOMER [22], Hi-Cdat [23] and HiC-Pro [24]. In the following, we demonstrate that the CGP map (*B*) can capture the correlation between spatial arrangement and co-regulation of genes in 3D better than the raw gene proximity map (*W*).

### CGP better encodes important functional and structural properties of the genome

**Fig. 2:**
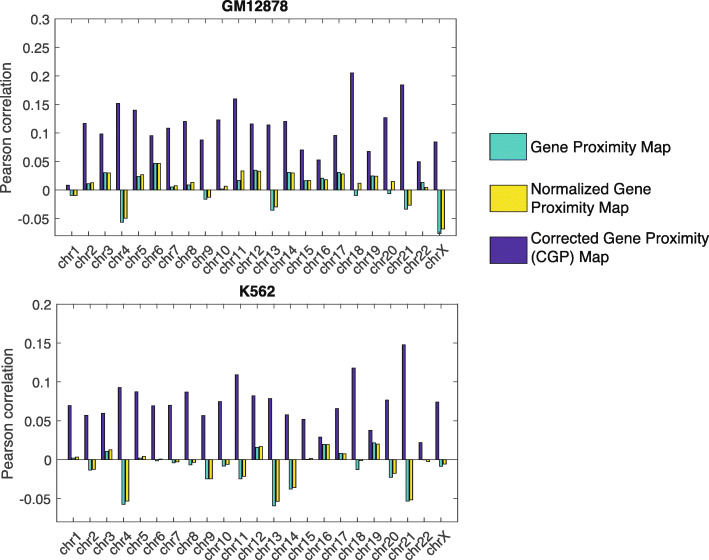
Pearson correlation coefficients between the gene co-expression matrix and three different matrices based on spatial positioning of genes: the CGP map (blue bars), the raw gene proximity map (green bars), and the normalized gene proximity map (yellow bars) for each of the 23 chromosomes in GM12878 cell line (top panel) and K562 cell line (bottom panel)

We investigated whether the CGP map can unravel functional properties of the genome such as gene co-expression. We used RNA-seq data and Hi-C data of 12 cell lines, including 10 cancer cell lines recently published by the ENCODE consortium [25], and two hematopoietic cell lines (GM12878 and K562) in which we have deeply sequenced Hi-C data [26] (see Methods for details). For each cell line and each chromosome, we used the RNA-seq data to build a co-expression matrix *C* (see Methods for details). Then, for each of the 23 chromosomes, we computed the Pearson correlation coefficient between *C* and two matrices: the CGP map *B* and the gene proximity map *W*. Figure 2 shows the results for GM12878 and K562 cell lines. For all 23 chromosomes: the correlation between *B* and *C* remains positive, whereas the correlation between *W* and *C* is low and alternates between positive and negative. Note that a normalized version of the gene proximity map $\overset{\sim }{W}$, similar to the normalized contact map introduced in [11], exhibits the same behavior as *W*. This analysis demonstrates that the CGP map is indeed better, information-wise, than the raw gene proximity map at explaining gene co-expression in the genome. Similar patterns can be observed in most of the other 10 cell lines (Fig. S1).

A common technical issue in contact map analysis is the diminishing read counts and thus statistical power as separation between loci increases. We investigated to what extent the CGP map could be limited by the issue. In particular, we checked whether the distant pairs dilute the correlation as observed in Fig. 2. As shown in Fig. S1B, by removing the distant pairs, we found that the correlation between co-expression matrix and the CGP map decreases, suggesting the distant pairs contribute to the signal of CGP.

It is well known that a genome could be divided into the so-called A/B compartments, in which compartment A tends to be active whereas compartment B consists of heterochromatin [2, 11]. Therefore, it is instructive to further examine whether the CGP map can capture such information. Motivated by the use of eigenvectors of the modularity matrix for community detection on networks, we formulated the problem as a classification problem and used the components of the leading eigenvectors of the CGP matrix as features (see Methods for details). We found that the CGP matrix works reasonably well in predicting to which compartment a gene belongs (AUC = 0.86, Fig. S2).

### CGP captures the interplay between gene spatial positioning and co-regulation

It has long been observed that co-expressed genes, or functionally related genes are likely to be next to each other on the 1D genome [27]. Recent studies further suggest that they are also spatially clustered in the nucleus [28]. Nevertheless, to what extent spatially clustered genes are co-expressed, and to what extent the tendency is attributed to various spatial organizations like compartments and TADs are not well characterized. Here, we quantify the interplay between the spatial positioning of genes and gene co-regulation by phrasing it as an optimization problem using the CGP map. More specifically, we assume the expression states of genes to be either ON (*x*_*i*_ =  + 1) or OFF (*x*_*i*_ =  − 1), and explore how the two states are distributed on the gene proximity network (see Methods for details). We define an objective function:

3 $$ Q=\frac{1}{\sum_{ij}{W}_{ij}}{\sum}_{ij}{B}_{ij}{x}_i{x}_j. $$

**Fig. 3:**
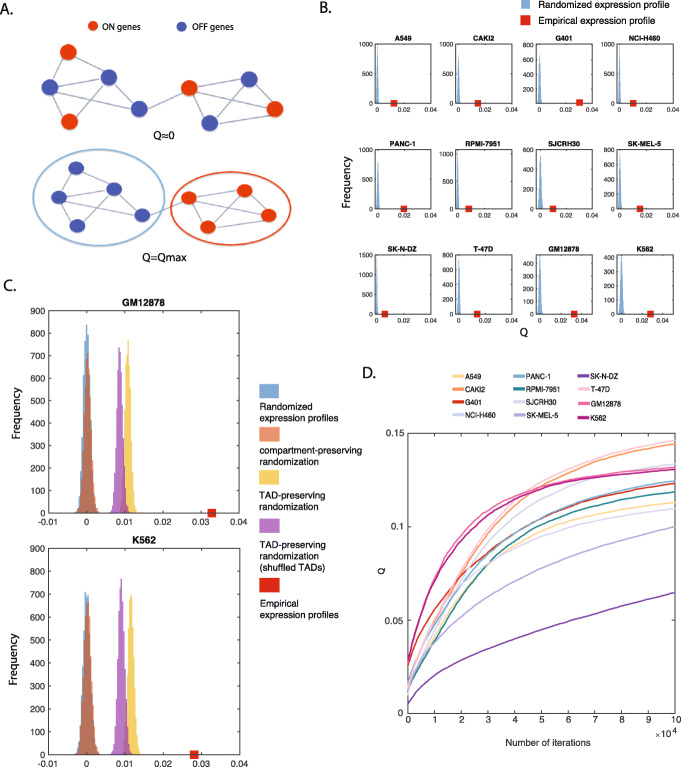
**a** An illustration of the CGP-based objective function *Q*: *Q* is maximum if ON genes and OFF genes are perfectly separated on the 3D genome; *Q* is close to zero if ON and OFF genes are randomly positioned. **b***Q* computed from the empirical gene expression profile and randomized profiles. The horizontal axis shows the value of the objective function and the vertical axis is the probability density function. The 12 subplots correspond to 12 individual cell lines: A549, CAKI2, G401, NCI-H460, PANC-1, RPMI-7951, SJCRH30, SK-MEL-5, SK-N-DZ, T-47D, GM12878 and K562 respectively. In each subplot, the red square indicates the value for the empirical profile, the histogram shows the distribution for values obtained from an ensemble of randomized profiles. **c** For cell lines GM12878 and K562, an additional ensemble of randomized profiles was generated by shuffling gene indices within TADs alone. The resultant distribution is shown in brown. **d** Optimization of *Q* using a Monte Carlo procedure. Starting from the empirical gene expression profile, there is room for increasing Q for each of the cell lines tested

Here, *Q* quantifies to what extent gene co-expression pattern is related to the underlying spatial proximity between genes. If there is no particular relationship between the spatial positioning of genes and their regulation, *Q* is close to 0 as genes with different expression states are randomly distributed in 3D (*B*_*ij*_ ≈ 0). On the other hand, if a large fraction of co-expressed genes are proximal (*B*_*ij*_ > 0), the overall value of *Q* is high (Fig. 3a). A similar objective function has previously been used to solve the classic network bisection problem [29].

As shown in Fig. 3b, in all 12 cell lines, the value of *Q* is much higher than 0. To show the statistical significance of the result, we looked at the distribution of *Q* in an ensemble of gene configurations where the spatial locations of genes are randomly redistributed. As expected, the values of *Q* generated from the random configurations appear to follow a Gaussian distribution with zero mean. In all tested cell types, the empirical expression profile yields a substantially greater function value, which is always positive, than the permuted profiles. The separation between the values of *Q* for the empirical configuration and random configurations is a measure of the tendency in which active genes tend to be proximal in space. We confirmed CGP indeed provides a better measure for such a tendency by repeating the analysis with a modified objective function computed using raw gene proximity map. Specifically, we found that the raw gene proximity map is less efficient at capturing the interplay between the spatial positioning of genes and co-regulation (Fig. S3).

We further explored to what extent the tendency is attributed to characterized spatial structures. For instance, it is well known that the genome is organized into A/B compartments. We therefore generated another ensemble by shuffling the genes in A/B compartments separately. We found that although the resultant distribution of *Q* is significantly higher than the null distribution (Figure 3c, *P* = 1.2 × 10^−21^ in GM12878 and *P* = 3.1 × 10^−19^ in K562), it is very much lower than the empirical *Q*. The analysis suggests that the compartment organization alone cannot account for the co-localization of expressed genes.

Genes within a compartment are further organized. Spatial structures of particular interest are the topologically associating domains [30, 31]. Topologically associating domains (TADs) are domains of self-interacting chromatin that have emerged as a fundamental structural unit in genome organization [32]. To investigate to what extent TADs contribute to the value of *Q*, we generated two more ensembles: One by shuffling the genes in TADs separately; the other one by permuting the TADs first and then shuffling the genes within the permuted TADs (Figure 3c). Since the permuted TADs are essentially random blocks with the same size distribution as the original TADs, the distribution of *Q* generated by this model in CGP quantifies the effect of genes from forming TADs in contrast to the effect of genes from simply being close in 1D sequence.

The different ensembles shown in Figure 3c take into account different levels of spatial organization. Since the null ensemble refers to a case in which genes are distributed with no regard to spatial structure, the separation between the null and the distribution of *Q* in the compartment-preserving ensemble or TAD-preserving ensemble correspond to the relative contribution of compartments or TADs. The observation that the empirical *Q* is higher than the distribution of TAD-preserving ensemble suggests genes are further organized within TADs in order to make co-expressed members proximal to each other. A natural question is, to what extent the co-expressed genes are organized favoring spatial proximity? To do this, we introduced a Monte Carlo procedure to iteratively search for the maximal value of *Q* (see Methods for details). More specifically, the positions of ON and OFF genes are swapped in order to increase the value of *Q*. As shown in Figure 3d, in all the tested CGP maps, given a fixed number of expressed genes, it is possible to further increase the value of *Q* from the empirical value, meaning that the empirical values are far from optimal.

### Change of CGP is highly correlated with gene expression change

**Fig. 4:**
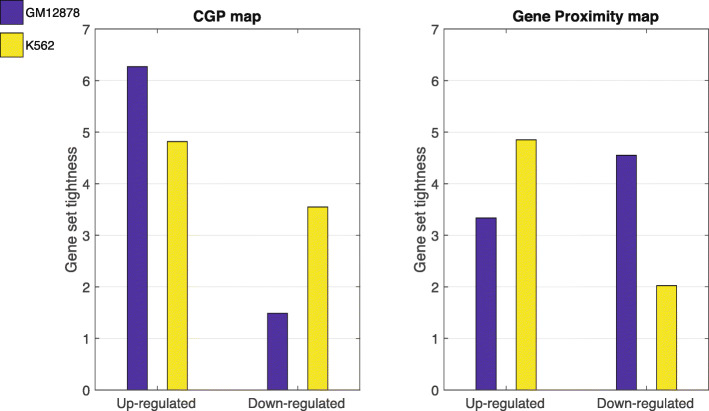
Gene set tightness for two groups of genes in GM12878 and K562 cell lines. The first group contains 100 most up-regulated genes in GM12878 as compared with K562 whereas the second group consists of 100 most down-regulated genes. From left to right, the gene set tightness is computed from the CGP map (*B*) and the gene proximity map (*W*), respectively. In both panels, the blue and yellow bars represent GM12878 and K562 cell lines, respectively. The gene set tightness quantifies to what extent a set of genes are spatially close together in the cell nucleus. A high tightness means the genes are spatially in closer proximity than expectation whereas a low tightness implies that genes are far apart

We further investigated whether there exists a correspondence between changes in expression profile and spatial configurations among different cell types. To do this, we focused on two hematopoietic cell lines: GM12878 and K562, where the latter is a cell line derived from a patient with chronic myeloid leukemia (CML). We considered two sets of genes based on differential expression: the first set contains the most up-regulated genes in GM12878 as compared with K562 (100 genes with the largest expression fold-change); the second set consists of the most down-regulated ones (100 genes with the smallest expression fold-change). We should expect that the first set of genes to be clustered more tightly in GM12878 than in K562, while the second set should be clustered more tightly in K562. Moreover, we would like to compare the effect measured on the CGP map with the one measured on the raw gene proximity map. We defined a quantity, *“tightness”*, denoted by *T*, to measure to what extent a set of genes is tightly positioned (i.e., the average spatial distance between genes is small) in 3D (see Methods for details). When computing *T* using the CGP, we found that the up-regulated genes in GM12878 are close together in GM12878 (high tightness) but farther apart (low tightness) in K562, while the down-regulated genes are more tightly clustered in K562 than in GM12878 (Figure 4, left panel). The right panel in Figure 4 shows that the result is not visible, when we obtain *T* using the gene proximity map alone. It contradicts our expectation in that the up-regulated genes, on average, have fewer contact frequencies between themselves than the down-regulated genes in a cell.

**Fig. 5:**
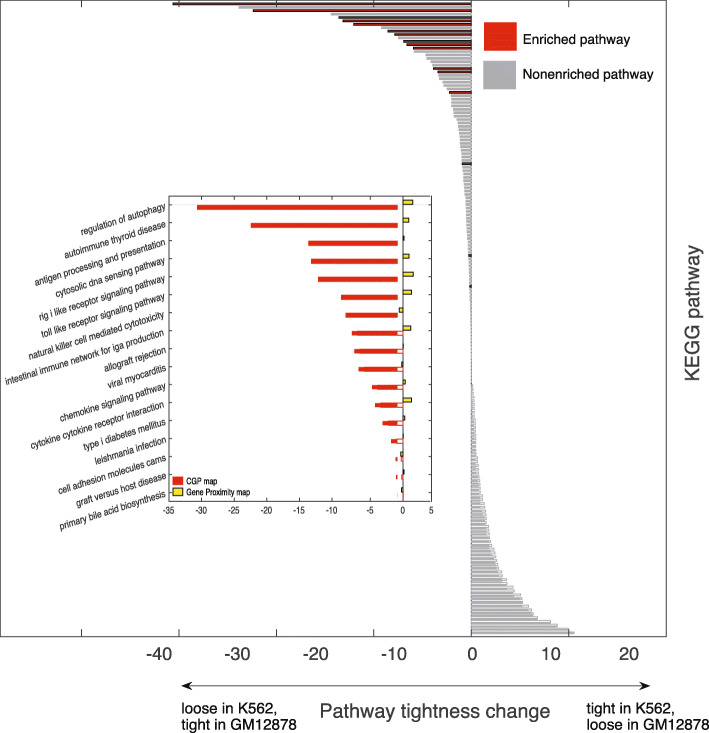
(Outset) Pathway tightness change, from cell lines GM12878 to K562, for all 186 metabolic pathways in the KEGG database. From top to bottom the pathways are sorted according to the tightness change computed from the CGP. The red bars represent the 17 pathways found to be statistically enriched with differentially expressed genes (up-regulated) in GM12878 with respect to K562. The horizontal axis shows the pathway tightness change. A negative value indicates that the pathway genes are in closer proximity in GM12878 but far apart in K562. A positive difference means the opposite. (Inset) Names and pathway tightness change of the 17 statistically enriched pathways. From top to bottom the pathways are ranked according to the tightness change computed from the CGP (red bars). The yellow bars represent the tightness change computed from the gene proximity map. The horizontal axis is the same as that in the outset

We performed the same analysis at the level of pathways. More specifically, we investigated how the spatial configuration of the genes constituting a pathway changes when their expression changes. For each of the 186 metabolic pathways in KEGG database, we calculated the difference in tightness between K562 and GM12878 cell lines. A positive (negative) difference indicates that genes in the pathway are tightly (loosely) positioned in K562 but loosely (tightly) positioned in GM12878. At the same time, we performed the Gene Set Enrichment Analysis (GSEA) and identified pathways that are enriched with genes that are up-regulated in GM12878 with respect to K562. As shown in Figure 5 (outset), we found that these up-regulated pathways are consistently more compact in GM12878 than in K562, which illustrates that, for these pathways to be expressed in GM12878 but not in K562, their genes tend to be spatially reorganized to achieve closer proximity. One example of such pathways is the regulation of autophagy, whose inhibition has been reported as a new strategy to induce cell death of drug-sensitive and drug-resistant CML cells [33]. This is probably due to the fact that autophagy genes are poorly expressed in K562 cells as compared with GM12878 cells and are therefore more sensitive to the inhibition of autophagy. Importantly, the figure shows that this effect is not visible when performing the same analysis using raw contact frequencies, as it fails to identify the correlation between the change in relative positioning of genes in 3D and the change in gene expression between cell lines (Figure 5, inset).

### CGP reveals critical inter-chromosomal interactions in the nucleus

The CGP map offers a natural framework to study the inter-chromosomal proximity between genes. Based on the values in the CGP matrices, we analyzed the 20 closest inter-chromosomal gene pairs in each of the K562 and GM12878 cell lines (Supplementary Materials Table S1). We observed that the pairs identified in two cell lines involve quite different sets of chromosomes, naming chr11-chr17 in GM12878 whereas chr9-chr13-chr22 in K562. This difference might due to the large-scale chromosomal rearrangement in the cancer cell line K562.

**Fig. 6:**
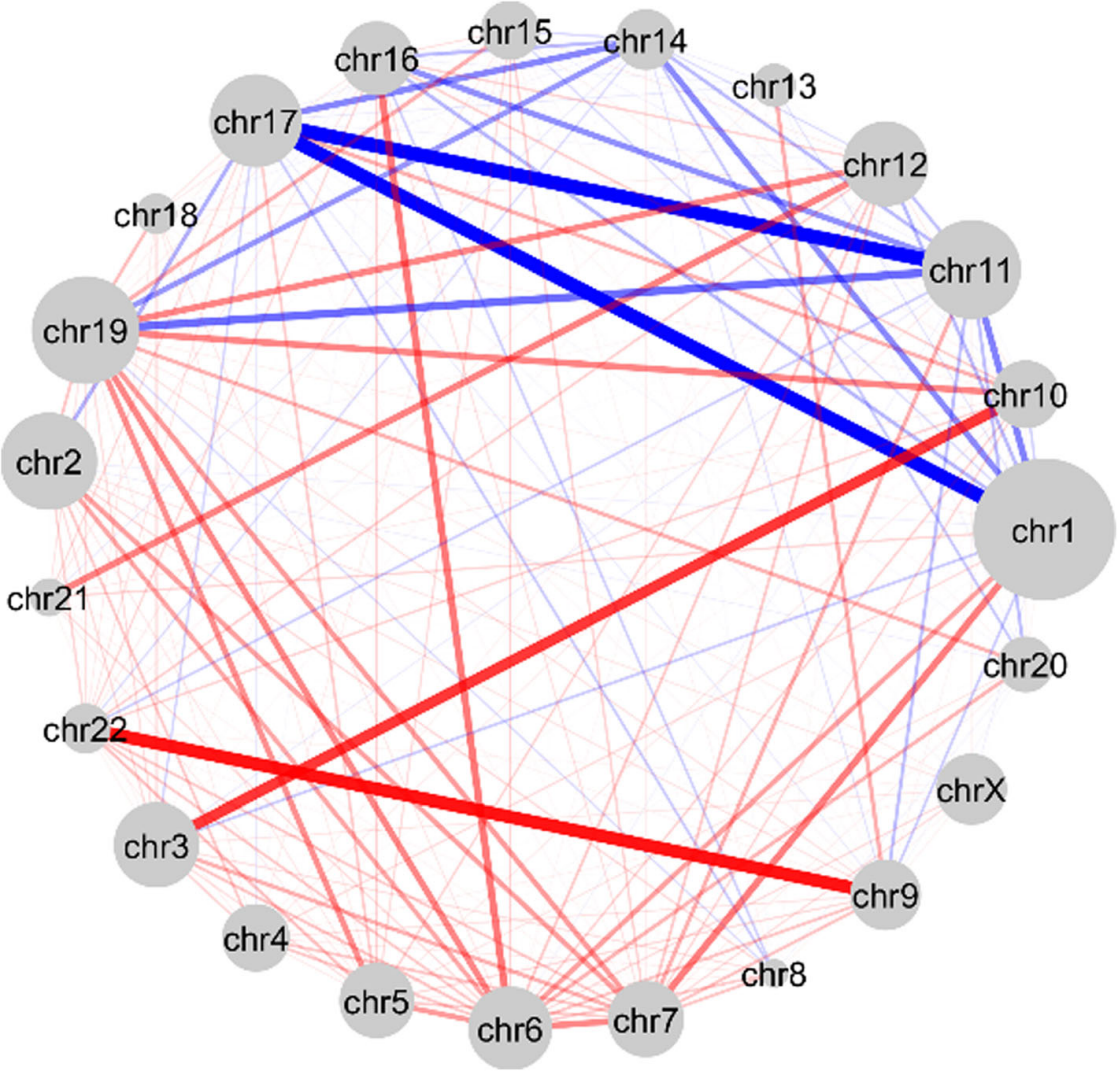
Change in relative spatial positioning of chromosomes between GM12878 and K562 cell lines. The 23 nodes correspond to 23 chromosomes. The node size is proportional to the number of genes in the chromosome. Weighted blue and red links illustrate the extent and direction of proximity changes. The thickness and color of an edge between nodes *i* and *j* represent the magnitude and sign of the inter-chromosomal corrected proximity difference from GM12878 to K562. A thick blue (red) edge means that the inter-chromosomal proximity is substantially smaller (greater) in K562 as compared with GM12878. The network is fully connected, many edges are not shown because their weights (change in corrected proximity) are too small

Apart from spatial proximity at gene level, it is interesting to investigate spatial proximity at chromosomal level. To do this, we applied a size-reduction technique [34] that merges genes belonging to the same chromosome in the CGP map together to form a reduced inter-chromosomal proximity map. Starting from the CGP matrix *B*, the corresponding *“inter-chromosomal proximity matrix”*$\hat{B}$ was obtained by merging the rows and columns according to the gene-chromosome correspondence (see Methods for details). We took CML as a case study here and compared the inter-chromosomal proximity map between GM12878 and K562 cell lines in order to obtain some insights on the role of abnormal chromosomal rearrangements in the diseased cell. Figure 6 summarizes the global change of spatial organization of the genome between K562 and GM12878 at the chromosome level. A positive change between a pair of chromosomes indicates that they are closer in 3D in K562 than in GM12878 and a negative change means the opposite. Weighted blue and red links illustrate the extent and direction of proximity changes: blue represents negative changes while red represents positive changes. The network recapitulates a number of known facts that are consistent with the genome of cells affected by CML. First, there is a red connection between chromosome 9 and 22, resembling the Philadelphia chromosome, a reciprocal translocation between chromosome 9 and chromosome 22 [35]. Second, chromosome 3 and 10 are linked by a red line, indicating this pair are closely located in the K562 cell line. This behavior was recently characterized as a rare chromosomal translocation in leukemia patients [36]. Both aforementioned translocations have been observed in the K562 cell line in a recent study [37]. As shown in Figure 6, chromosome 17 appears to move away from several chromosomes including 1, 11, 14 and 19. This could be attributed to an abnormality described as the single most important cytogenetic abnormality for the prognosis of leukemia [38]. In short, we found the inter-chromosomal proximity map, derived from the CGP map, captures the relative spatial organization of chromosomes between cell types. With no surprise, the critical chromosomal abnormalities cannot be effectively identified using the gene proximity map *W* (Figure S4).

## Discussion

In this study, we have proposed a network framework for studying the 3D structure of the genome on a global scale. As recognized by previous studies, the gene contact frequency signal consists of the 1D component and 3D component, where the 1D component is the expected number of contacts between genes based exclusively on their genomic distance. At the heart of our approach is a mathematical framework that separates the 1D component of the genome and embeds the 3D component into the CGP map. Since all cells in the human body essentially have an identical 1D genome, the extracted CGP map therefore encodes cell specific arrangements of genes in 3D, which reflects the cell type specific role in genome functions. Compared with the gene proximity map, the CGP map can be viewed as a “signal-separated” version that models the underlying spatial positioning of genes in 3D.

We have shown that the CGP map can reveal genome functional properties, such as co-expression, more efficiently, while preserving genome structural information, e.g., compartments, contained in the Hi-C result. We have found that the change of gene expression in relation with spatial positioning can be effectively captured by the CGP. Moreover, by using a size-reduction procedure, the CGP map can effectively identify critical differences in inter-chromosomal interactions between cell types. Though it is well known that co-expressed genes have a tendency to be spatially close together in the 3D genome, the three-dimensional placement of genes in proximity to shared transcriptional machinery within the nucleus can be thought of as a cell type specific program. We have formulated an optimization framework to quantify the interplay between gene expression and spatial proximity based on the CGP, and in particular to analyze to what extent a particular spatial structure plays a role for the tendency. In fact, the tendency that co-expressed genes are spatially close together in the genome suggests certain selective advantages. For instance, it is possible that bringing genes together makes a more efficient use of the transcription machinery.

Network modeling serves as a flexible framework to organize biological data [39]. The CGP map is based on a network analysis framework. Over the last decades, network approaches, e.g., protein-protein interaction network analysis, have been widely used in studying the structure of many biological entities such as proteins [40–42]. This has inspired scientists to use these tools to analyze a different but conceptually related problem, that of the structure of the whole genome. Recent studies have shown that network theory has many useful applications in the study of Hi-C contact maps, e.g., the similarity between the spectral properties of two contact maps quantifies the reproducibility of two Hi-C experiments [43]. More recently, network modularity has been used in intra-chromosomal contact maps to identify TADs [44, 45]. In fact, the developed CGP map can be thought of as a mathematical generalization of the modularity matrix in network theory [46]. In this study, we have restricted our analysis to protein-coding genes. It has been shown that non-coding RNAs can exploit the 3D organization of the genome for their function, for instance, Xist in X activation [47]. Our framework can also be easily generalized to include non-coding elements. Apart from expression data, we could easily include other genomic features such as transcription factor binding sites, mutation rates, and histone modifications onto the network as the properties of nodes. A variety of biological problems can then be set up in the CGP. For instance, examining the clustering of RNA polymerase binding sites in the network is closely related to the search of transcription factories [48]. A size-reduction approach like the one shown to reduce the gene proximity network into an inter-chromosomal proximity network could be employed to study the hierarchical organization of the 3D genome, for instance, chromosome territories [49].

## Methods

### Hi-C data source and pre-processing

The ENCODE Hi-C data were released by the ENCODE consortium [25]. Ten cell lines are used in the analysis, including T47D, A549, Caki2, G401, NCI-H460, Panc-1, RPMI-7951, SJCRH30, SK-N-DZ and SK-MEL-5. For each cell line, two replicates are separately used. The ENCODE Hi-C data are processed by the tool cworld (https://github.com/dekkerlab/cworld-dekker). Hi-C data of K562 and GM12878 cell lines were reported in [11]. For ENCODE Hi-C contact maps, a bin size of 40 kb is used, whereas the bin size of K562 and GM12878 Hi-C contact maps is 25 kb. The whole-genome contact maps of all cell lines studied have been iteratively corrected for uniform coverage by the ICE algorithm [21].

### Construction of the gene proximity network

The gene proximity network is obtained by extracting elements from the genome-wide Hi-C contact map. Denote *W* as the weighted network, whereas *i* and *j* index two genes. The matrix element *W*_*ij*_ is determined by mapping the genomic coordinates of genes *i* and *j* to the corresponding genome-wide contact map. If each gene is located within a single bin in the contact map (bin sizes of 25 kb or 40 kb were used in this study), *W*_*ij*_ is chosen to be the corresponding contact frequency between the two bins in the contact map. If either gene spans across multiple bins, the maximum contact frequency among the bins is chosen to represent the minimum distance between genes. The normalized gene proximity map $\overset{\sim }{W}$ is defined by dividing each entry in the gene proximity map by the expected number of contacts between those two genes, i.e., ${\overset{\sim }{W}}_{ij}=\frac{W_{ij}}{E_{ij}}$.

### Null model for the 1D component of the gene contact frequency signal

Given a gene proximity network, which is derived from the genome-wide Hi-C contact map for a cell line, a corresponding null model for the 1D component of the gene contact frequency is defined. To define the null model, a cell type specific function *f*(*d*) that maps the genomic distance *d* between a pair of genes to a real value *f* is estimated from the genome-wide Hi-C contact map. In this study, *d* is measured in the unit of the bin size of the contact map.

If gene *i* and gene *j* are on the same chromosome, separated by a genomic distance *d*, *f*(*d*_*ij*_) is defined as *f*^*intra*^(*d*), which is then estimated by the average of the contact frequencies over all pairs of genomic loci that are separated by *d* in the genome-wide Hi-C contact map, with the employment of a local smoothing approach similar to the one used in [50]. *f*^*intra*^(*d*) is therefore a monotonically decreasing function, which means that genes next to each other on the chromosome are more likely to interact whereas genes far apart are less likely to interact. On the other hand, if gene *i* and gene *j* are on different chromosomes, *f*(*d*_*ij*_) is *f*^*inter*^, a constant estimated by the average of all inter-chromosomal interactions between genomic loci in the genome-wide contact map. This reflects the idea that all the genes locating on one chromosome have equal chances to interact with all the genes on the other.

Given the estimated function *f*(*d*), the expected number of contacts between gene *i* and gene *j* is defined by Equation (1). To obtain the values for *k*_*i*_, Equations (1) and (2) are rewritten in the form:

4 $$ {\sum}_j{k}_i{k}_jf\left({d}_{ij}\right)={\sum}_j{W}_{ij}. $$

This system of nonlinear equations is then solved by an iterative procedure described in [44] where *k*_*i*_ is initialized as $\sqrt{\sum_j{W}_{ij}}$, and after successive iterations and normalization, converged to the solution of Equation (4).

The code for the entire pipeline for generating the CGP map from Hi-C data is available at http://www.paccanarolab.org/cgp/.

### Gene expression data

Gene expression data of all cell lines used in this study were downloaded from the ENCODE portal (https://www.encodeproject.org/). The gene co-expression matrix *C* of a cell line is computed via the corresponding gene expression data: the (*i*, *j*)-th entry in *C* is the product of the (logarithm) expression value of genes *i* and *j*. Genes with zero expression level are manually set to have very small (negative) values. So, a positive value indicates that two genes have similar expression states (either both active or both inactive) whereas a negative value implies they have opposite states (one active, one inactive).

In the gene expression and CGP interplay analysis, genes are binarized into ON and OFF states for each individual cell line. In particular, a Gaussian mixture model clustering algorithm is applied on the (logarithm) gene expression profile in order to identify two gene clusters. The cluster with the higher average expression value is considered as ON genes whereas the other cluster corresponds to OFF genes.

### Genome compartment data

The genome compartment data of GM12878 and K562 cell lines were downloaded from [11]. The compartments were defined based on the first principal component of the normalized contact matrix: positive and negative entries correspond to A and B compartment, respectively. All genes were then assigned to either A or B compartment based on their genomic coordinates. Predicting the compartment label of a gene was formulated as a binary classification problem. In particular, the components of the 50 leading eigenvectors of the CGP matrix were used as features. An ensemble of bagged decision trees was trained to solve this binary classification problem. The optimal model parameters, e.g., the maximum number of splits and the number of learners, were tuned via a standard 10-fold cross-validation. All the model learning procedures were implemented in MATLAB R2018a using the Statistics and Machine Learning Toolbox.

### Monte Carlo optimization

A Monte Carlo approach was used to investigate if the empirical expression is optimal with respect to the structure of the weighted gene proximity network. Starting with the empirical gene expression profile, for each step, we randomly picked a pair of genes and swapped their location in the network. The swapping was kept if the value of the objective function was increased and ignored otherwise.

### Gene set tightness

Given a set of genes *p*, an unnormalized tightness measure (i.e., how well these genes are closely positioned in 3D) can be calculated by summing over elements in the CGP map for every pair of genes:

5 $$ {b}_p={\sum}_{i,j\in p}{B}_{ij}. $$

To estimate the statistical significance of *b*_*p*_ and obtain a normalized tightness, 5000 randomized gene sets are generated. A random counterpart for gene set *p* is obtained by selecting *n*_*p*_ (the number of genes in *p*) genes uniformly at random from all genes. By calculating the overall corrected proximity for each of the randomized counterparts, a null distribution for the overall corrected proximity of *p* is estimated. The tightness of *p* is then defined as the deviation of *b*_*p*_ from the null distribution, i.e., *T*_*p*_ = (*b*_*p*_ − *μ*_*p*_)/*σ*_*p*_, where *μ*_*p*_ and *σ*_*p*_ are the mean and standard deviation of the null distribution. A very positive *T*_*p*_ suggests that the genes in *p* are substantially closer to each other than average, whereas a very negative *T*_*p*_ indicates that the physical distance between those genes is dramatically greater than that of randomly selected genes.

### Inter-chromosomal proximity map

Starting from the CGP matrix *B*, the corresponding inter-chromosomal proximity matrix $\hat{B}$ is obtained by merging the rows and columns according to the gene-chromosome correspondence. The element ${\hat{B}}_{\alpha \beta}$, which represents the inter-chromosomal proximity between chromosome *α* and chromosome *β* (*α* ≠ *β*), is computed as follows

6 $$ {\hat{B}}_{\alpha \beta}={\sum}_{i\in \alpha, j\in \beta }{B}_{ij}\cdot $$

$\hat{B}$ is therefore a 23-by-23 square matrix where the main diagonal are all zeros and the inter-chromosomal proximity elsewhere. This matrix is then normalized with respect to its Frobenius norm, i.e., $\overset{\sim }{B}=\hat{B}/{\left\Vert \hat{B}\right\Vert}_F,$ so that $\overset{\sim }{B}$ derived from different cell types are comparable.

## Supplementary information

**Additional file 1: Figure S1.** Pearson correlation coefficients between the gene co-expression matrix and three different matrices based on spatial positioning of genes: the CGP map (blue bars), the raw gene proximity map (green bars), and the normalized gene proximity map (yellow bars) for each of the 23 chromosomes for 10 ENCODE cell lines. **Figure S2.** (A) ROC curve for the gene compartment classification using leading eigenvectors of the CGP matrix for GM12878 and K562 cell lines. The horizontal axis is the false positive rate (1 − specificity) and the vertical axis is the true positive rate (sensitivity). The red dot indicates the optimal operating point. Components of the top 50 leading eigenvectors were used as features for the classification model. (B) Effect of the number of eigenvectors used in the gene compartment label classifier. The horizontal axis represents the number of eigenvectors in the CGP matrix used for model construction, ranged from 1 to 50. The vertical axis is the average AUROC of the resultant model over the 10-fold cross validation. The red circles and blue squares (almost completely coincide) represent the GM12878 and K562 cell lines respectively. Using the first leading eigenvector alone does not yield a good classification result. By additionally incorporating the second and third eigenvectors, the AUROC witnesses a dramatic increase (from 0.57 to 0.70). On the other hand, using more than 10 eigenvectors does not provide a substantial performance improvement any more. **Figure S3.** Objective function based on the empirical gene expression profile and randomized profiles, computed using the raw gene proximity map. The histogram for randomized profiles is normalized to have zero mean. A main difference between the plots generated from the CGP and the raw gene proximity map is that for cell lines RPMI-7951, SJCRH30 and SK-N-DZ, the value of the gene proximity map-based objective function generated from the empirical expression profile is mixed with the values generated from randomized profiles. **Figure S4.** Change in relative spatial positioning of chromosomes between cell lines GM12878 and K562. The layout of this network is in the same way as Figure 6 in the main text, but the inter-chromosomal proximity matrix here was computed using the gene proximity map instead of the corrected proximity measure. As compared to Figure 6, the connections between chromosomes 3 and 10, and between chromosomes 9 and 22, are no longer easily identified. **Table S1**. Top 20 inter-chromosomal gene interactions in cell lines GM12878 and K562 respectively. These pairs of genes were selected based on the fact that they are located on different chromosomes and have the largest values in the corresponding CGP map.

## Data Availability

All Hi-C data analyzed during this study are included in [11, 25] (and their supplementary information files). The code for the entire pipeline for generating the CGP map from Hi-C data is available at http://www.paccanarolab.org/cgp/.

## Abbreviations

CGP: corrected gene proximity
RNA-seq: RNA sequencing;
AUC: area under curve
TAD: topologically associated domain
CML: chronic myeloid leukemia
GSEA: gene set enrichment analysis.
