# Supplementary material for: The corrected gene proximity map for analyzing the 3D genome organization using Hi-C data
Source: BMC Bioinformatics. 2020 May 29;21:222. doi: 10.1186/s12859-020-03545-y (PMC7260828; doi:10.1186/s12859-020-03545-y)
Supplement: Supplementary file 1 — Additional file 1: Figure S1. Pearson correlation coefficients between the gene co-expression matrix and three different matrices based on spatial positioning of genes: the CGP map (blue bars), the raw gene proximity map (green bars), and the normalized gene proximity map (yellow bars) for each of the 23 chromosomes for 10 ENCODE cell lines. Figure S2. (A) ROC curve for the gene compartment classification using leading eigenvectors of the CGP matrix for GM12878 and K562 cell lines. The horizontal axis is the false positive rate (1 − specificity) and the vertical axis is the true positive rate (sensitivity). The red dot indicates the optimal operating point. Components of the top 50 leading eigenvectors were used as features for the classification model. (B) Effect of the number of eigenvectors used in the gene compartment label classifier. The horizontal axis represents the number of eigenvectors in the CGP matrix used for model construction, ranged from 1 to 50. The vertical axis is the average AUROC of the resultant model over the 10-fold cross validation. The red circles and blue squares (almost completely coincide) represent the GM12878 and K562 cell lines respectively. Using the first leading eigenvector alone does not yield a good classification result. By additionally incorporating the second and third eigenvectors, the AUROC witnesses a dramatic increase (from 0.57 to 0.70). On the other hand, using more than 10 eigenvectors does not provide a substantial performance improvement any more. Figure S3. Objective function based on the empirical gene expression profile and randomized profiles, computed using the raw gene proximity map. The histogram for randomized profiles is normalized to have zero mean. A main difference between the plots generated from the CGP and the raw gene proximity map is that for cell lines RPMI-7951, SJCRH30 and SK-N-DZ, the value of the gene proximity map-based objective function generated from the empirical expression profile is mixed [file 12859_2020_3545_MOESM1_ESM.zip › Supplementary_material.pdf]

Supplementary Materials for "The Corrected Gene Proximity map for embedding the 3D genome organization in a graph using Hi-C data"

Cheng Ye[1], Alberto Paccanaro[1,2*], Mark Gerstein[3], Koon-Kiu Yan[4*]

[1]Department of Computer Science, Centre for Systems and Synthetic Biology, Royal Holloway, University of London, Egham, TW20 0EX, UK

[2]School of Applied Mathematics, Fundação Getulio Vargas, Rio de Janeiro, Brazil

[3]Program in Computational Biology and Bioinformatics, Department of Molecular Biophysics and Biochemistry, Department of Computer Science, Department of Statistics and Data Science, Yale University, New haven, CT 06520, USA

[4]Department of Computational Biology, St. Jude Children's Research Hospital, Memphis, TN 38105-3678, USA

[*]Corresponding author. E-mail: alberto.paccanaro@rhul.ac.uk; koon-kiu.yan@stjude.org

S1. CGP matrix can be thought of as a generalized modularity matrix for the gene proximity network

Many real-world networks display a specific structural feature, namely modules (or communities, clusters, etc.), where the network nodes in the same module are densely connected and where nodes in different modules are loosely connected [1]. To quantitatively assess to what extent a network can be divided into modules, an appropriate null model is required. A typical null model widely used in network analysis is the randomized network, a network with edges shuffled but that preserves the degree distribution of the original. Mathematically, given an unweighted, undirected network with adjacency matrix A, the randomized network is represented by a matrix R in which

$$R_{ij} = \frac{k_i k_j}{2m}.$$

R_{ij} estimates the expected number of edges between a pair of nodes i and j, if all the edges in the network (m in total) were randomly placed between nodes based on their node degrees k_i and k_j. Importantly, such a definition guarantees that the randomized network preserves the raw node degree distribution observed in A.

The null model E defined in Equation (1) in the main text can be thought of as a generalized version of the randomized network model R. In fact, E_{ij} estimates the expected number of contacts between a pair of genes and k_i can be thought of as a modified "node degree" that represents the visibility of a gene. Also, E takes the same form as R when $f(d_{ij}) = \frac{1}{2m}$, i.e., the expected number of contacts between two genes is simply proportional to the product of their intrinsic visibility, regardless of their locations on the 1D genome. Apparently, compared with R, E is an improved randomized model, as it is based on the assumption that the contact frequency between genes depends on both gene visibility and genomic distance. Following the conventional definition of the modularity matrix, the CGP map, defined as $B = W - E$, can thus be thought of as a generalized and improved modularity matrix for the gene proximity network.

S2. Cluster detection on the gene proximity network using the CGP matrix

Earlier Hi-C experiments showed that the genome can be segregated into two spatial compartments, namely A/B compartments, where regions in the same compartment tend to interact preferentially and where the inter-compartmental interactions are less likely to occur [2, 3]. In terms of the gene proximity network, this can be formulated as a network bisection problem in which the network is segregated into exactly two communities where the overall spatial distance between genes belonging to the same community is small, and where genes belonging to different communities are far apart.

An effective strategy for bisecting a network is to exploit the leading eigenvector of the network modularity matrix [4]. In particular, it has been shown that the modularity matrix plays the same role in maximizing the modularity index as that played by Laplacian matrix in spectral partitioning [5]. Similar to

standard spectral partitioning, the sign of the entry in the leading eigenvector of the modularity matrix determines an effective network division: nodes with positive and negative entries are assigned to their clusters, respectively. Since the CGP matrix can be thought of as a modularity matrix for the gene proximity network, its leading eigenvectors can be effectively used for detecting the two gene clusters.

Figure S1

Pearson correlation coefficients between the gene co-expression matrix and three different matrices based on spatial positioning of genes: the CGP map (blue bars), the raw gene proximity map (green bars), and the normalized gene proximity map (yellow bars) for each of the 23 chromosomes for 10 ENCODE cell lines.

Figure S2

(A) ROC curve for the gene compartment classification using leading eigenvectors of the CGP matrix for GM12878 and K562 cell lines. The horizontal axis is the false positive rate (1 − specificity) and the vertical axis is the true positive rate (sensitivity). The red dot indicates the optimal operating point. Components of the top 50 leading eigenvectors were used as features for the classification model. (B) Effect of the number of eigenvectors used in the gene compartment label classifier. The horizontal axis represents the number of eigenvectors in the CGP matrix used for model construction, ranged from 1 to 50. The vertical axis is the average AUROC of the resultant model over the 10-fold cross validation. The red circles and blue squares (almost completely coincide) represent the GM12878 and K562 cell lines respectively. Using the first leading eigenvector alone does not yield a good classification result. By additionally incorporating the second and third eigenvectors, the AUROC witnesses a dramatic increase (from 0.57 to 0.70). On the other hand, using more than 10 eigenvectors does not provide a substantial performance improvement any more.

Figure S3

Objective function based on the empirical gene expression profile and randomized profiles, computed using the raw gene proximity map. The histogram for randomized profiles is normalized to have zero mean. A main difference between the plots generated from the CGP and the raw gene proximity map is that for cell lines RPMI-7951, SJCRH30 and SK-N-DZ, the value of the gene proximity map-based objective function generated from the empirical expression profile is mixed with the values generated from randomized profiles.

Figure S4

Change in relative spatial positioning of chromosomes between cell lines GM12878 and K562. The layout of this network is in the same way as Figure 6 in the main text, but the inter-chromosomal proximity matrix here was computed using the gene proximity map instead of the corrected proximity measure. As compared to Figure 6, the connections between chromosomes 3 and 10, and between chromosomes 9 and 22, are no longer easily identified.

Table S1

Top 20 inter-chromosomal gene interactions in cell lines GM12878 and K562 respectively. These pairs of genes were selected based on the fact that they are located on different chromosomes and have the largest values in the corresponding CGP map.

GM12878

Gene 1	Chromosome	Gene 2	Chromosome
**PRSS1**	chr7	**PRSS3**	chr9
**DOC2B**	chr17	**SCGB1C1**	chr11
**DOC2B**	chr17	**ODF3**	chr11
**BET1L**	chr11	**DOC2B**	chr17
**BTRC**	chr10	**NBR1**	chr17
**BTRC**	chr10	**TMEM106A**	chr17
**NBR1**	chr17	**SLC3A2**	chr11
**SLC3A2**	chr11	**TMEM106A**	chr17
**NBR1**	chr17	**WDR74**	chr11
**TMEM106A**	chr17	**WDR74**	chr11
**KMT2C**	chr7	**TPTE**	chr21
**FRG1**	chr4	**FRG1B**	chr20
**RPH3AL**	chr17	**SCGB1C1**	chr11
**ODF3**	chr11	**RPH3AL**	chr17
**BET1L**	chr11	**RPH3AL**	chr17
**CWH43**	chr4	**DEFB115**	chr20
**FAM57A**	chr17	**SCGB1C1**	chr11
**GEMIN4**	chr17	**SCGB1C1**	chr11

**SCGB1C1**	chr11	**VPS53**	chr17
**FAM57A**	chr17	**ODF3**	chr11

K562

Gene 1	Chromosome	Gene 2	Chromosome
**ABL1**	chr9	**BCR**	chr22
**ABL1**	chr9	**RAB36**	chr22
**ABL1**	chr9	**RTDR1**	chr22
**ABL1**	chr9	**GNAZ**	chr22
**BCR**	chr22	**QRFP**	chr9
**QRFP**	chr9	**RAB36**	chr22
**BCR**	chr22	**FIBCD1**	chr9
**FIBCD1**	chr9	**RAB36**	chr22
**QRFP**	chr9	**RTDR1**	chr22
**GNAZ**	chr22	**QRFP**	chr9
**FIBCD1**	chr9	**RTDR1**	chr22
**FIBCD1**	chr9	**GNAZ**	chr22
**BCR**	chr22	**LAMC3**	chr9
**LAMC3**	chr9	**RAB36**	chr22
**LAMC3**	chr9	**RTDR1**	chr22
**GNAZ**	chr22	**LAMC3**	chr9
**FAM155A**	chr13	**FAM78A**	chr9
**FAM155A**	chr13	**NUP214**	chr9

**AIF1L**	chr9	**BCR**	chr22
**AIF1L**	chr9	**RAB36**	chr22

References

1. Newman, M.E.J., *Modularity and Community Structure in Networks.* Proceedings of the National Academy of Sciences, 2006. **103**(23): p. 8577-8582.
2. Lieberman-Aiden, E., et al., *Comprehensive Mapping of Long-Range Interactions Reveals Folding Principles of the Human Genome.* Science, 2009. **326**(5950): p. 289-293.
3. Rao, S.S., et al., *A 3D map of the human genome at kilobase resolution reveals principles of chromatin looping.* Cell, 2014. **159**(7): p. 1665-80.
4. Newman, M.E.J., *Spectral methods for community detection and graph partitioning.* Physical Review E, 2013. **88**(4): p. 042822.
5. Newman, M.E.J., *Finding community structure in networks using the eigenvectors of matrices.* Physical Review E, 2006. **74**(3): p. 036104.
