# Supplementary figures and images for: The corrected gene proximity map for analyzing the 3D genome organization using Hi-C data
Source: BMC Bioinformatics. 2020 May 29;21:222. doi: 10.1186/s12859-020-03545-y (PMC7260828; doi:10.1186/s12859-020-03545-y)

A

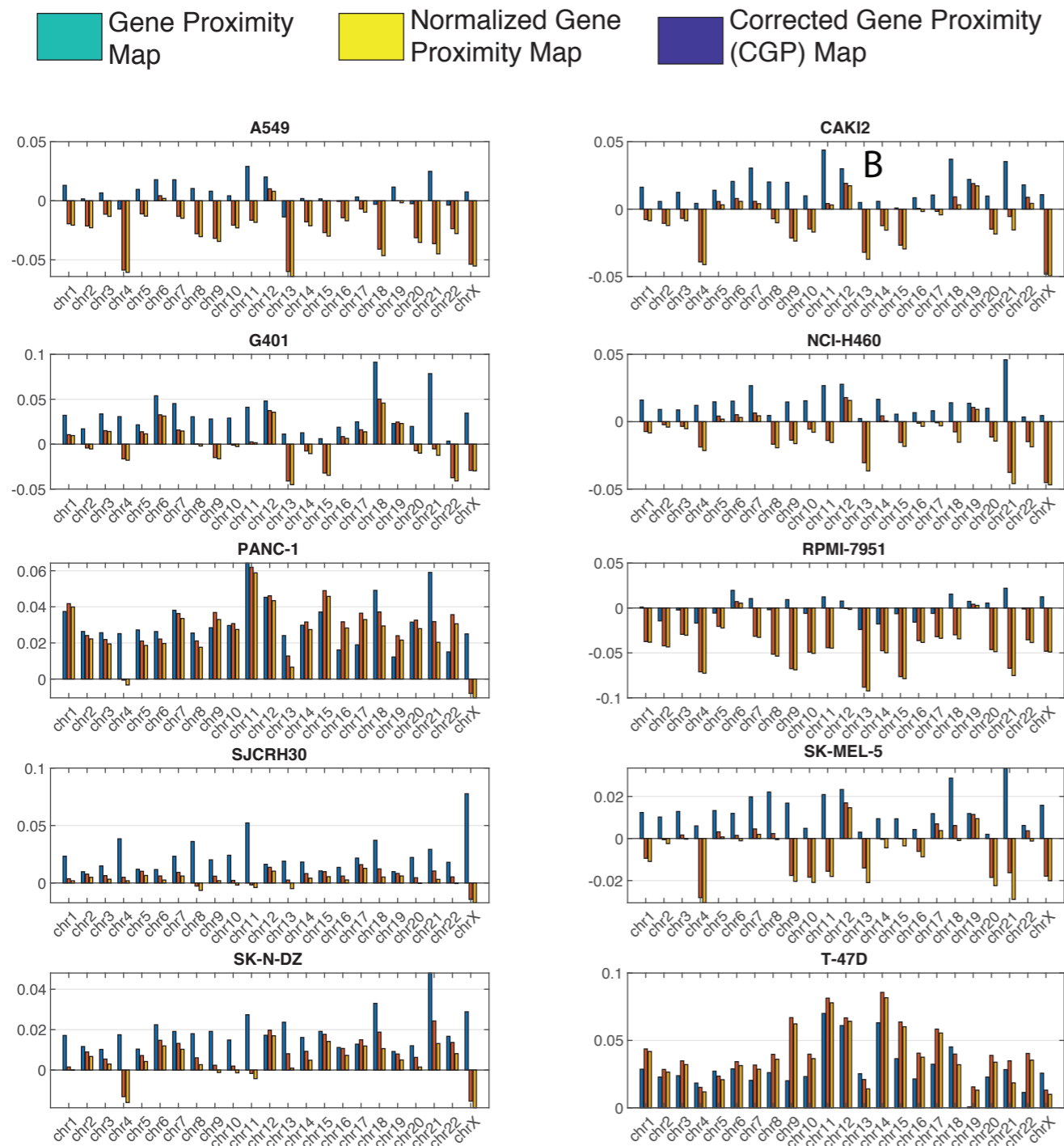

B

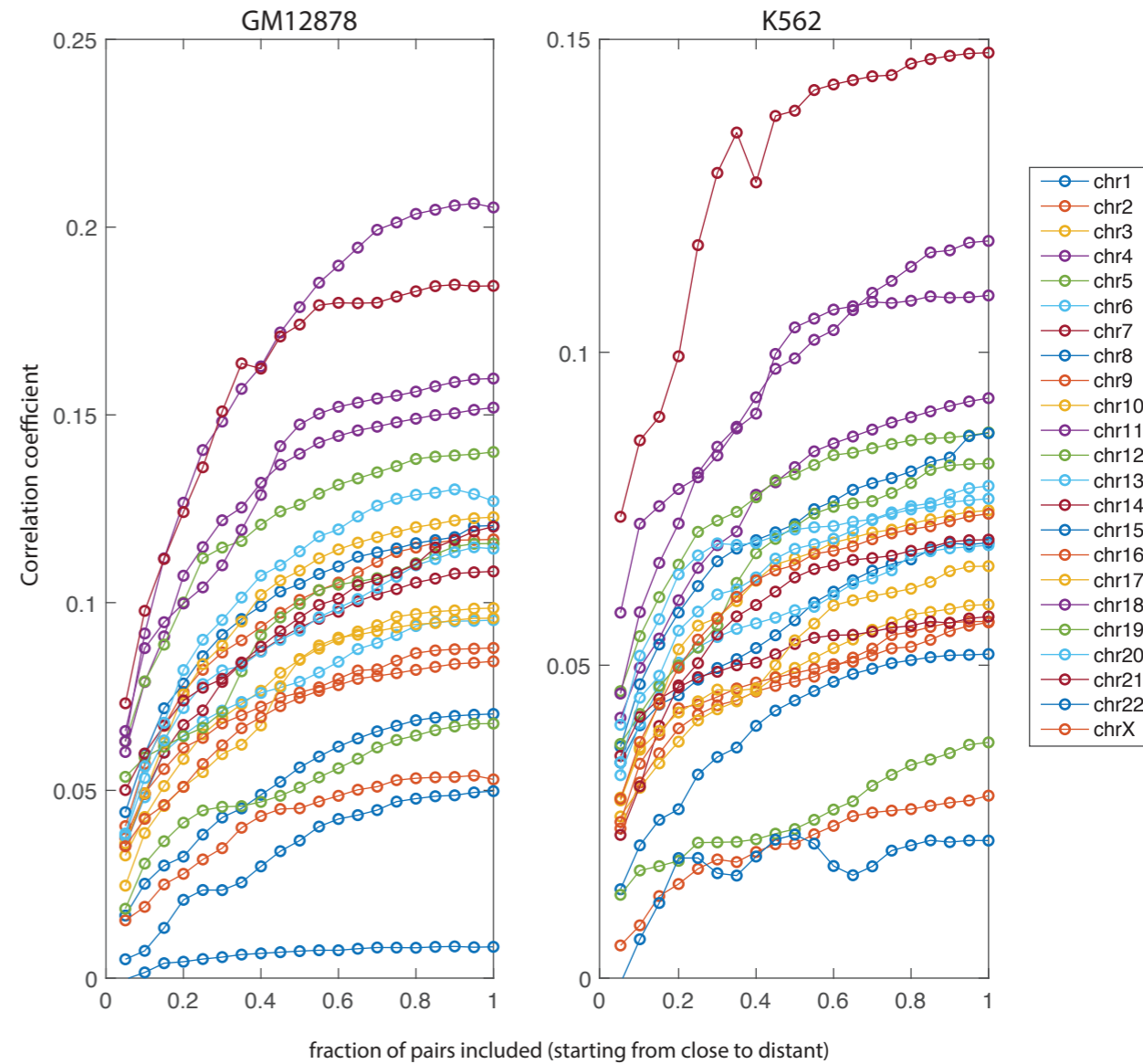

Supplement: Supplementary file 1 — Additional file 1: Figure S1. Pearson correlation coefficients between the gene co-expression matrix and three different matrices based on spatial positioning of genes: the CGP map (blue bars), the raw gene proximity map (green bars), and the normalized gene proximity map (yellow bars) for each of the 23 chromosomes for 10 ENCODE cell lines. Figure S2. (A) ROC curve for the gene compartment classification using leading eigenvectors of the CGP matrix for GM12878 and K562 cell lines. The horizontal axis is the false positive rate (1 − specificity) and the vertical axis is the true positive rate (sensitivity). The red dot indicates the optimal operating point. Components of the top 50 leading eigenvectors were used as features for the classification model. (B) Effect of the number of eigenvectors used in the gene compartment label classifier. The horizontal axis represents the number of eigenvectors in the CGP matrix used for model construction, ranged from 1 to 50. The vertical axis is the average AUROC of the resultant model over the 10-fold cross validation. The red circles and blue squares (almost completely coincide) represent the GM12878 and K562 cell lines respectively. Using the first leading eigenvector alone does not yield a good classification result. By additionally incorporating the second and third eigenvectors, the AUROC witnesses a dramatic increase (from 0.57 to 0.70). On the other hand, using more than 10 eigenvectors does not provide a substantial performance improvement any more. Figure S3. Objective function based on the empirical gene expression profile and randomized profiles, computed using the raw gene proximity map. The histogram for randomized profiles is normalized to have zero mean. A main difference between the plots generated from the CGP and the raw gene proximity map is that for cell lines RPMI-7951, SJCRH30 and SK-N-DZ, the value of the gene proximity map-based objective function generated from the empirical expression profile is mixed [file 12859_2020_3545_MOESM1_ESM.zip › FigureS1.pdf]

A

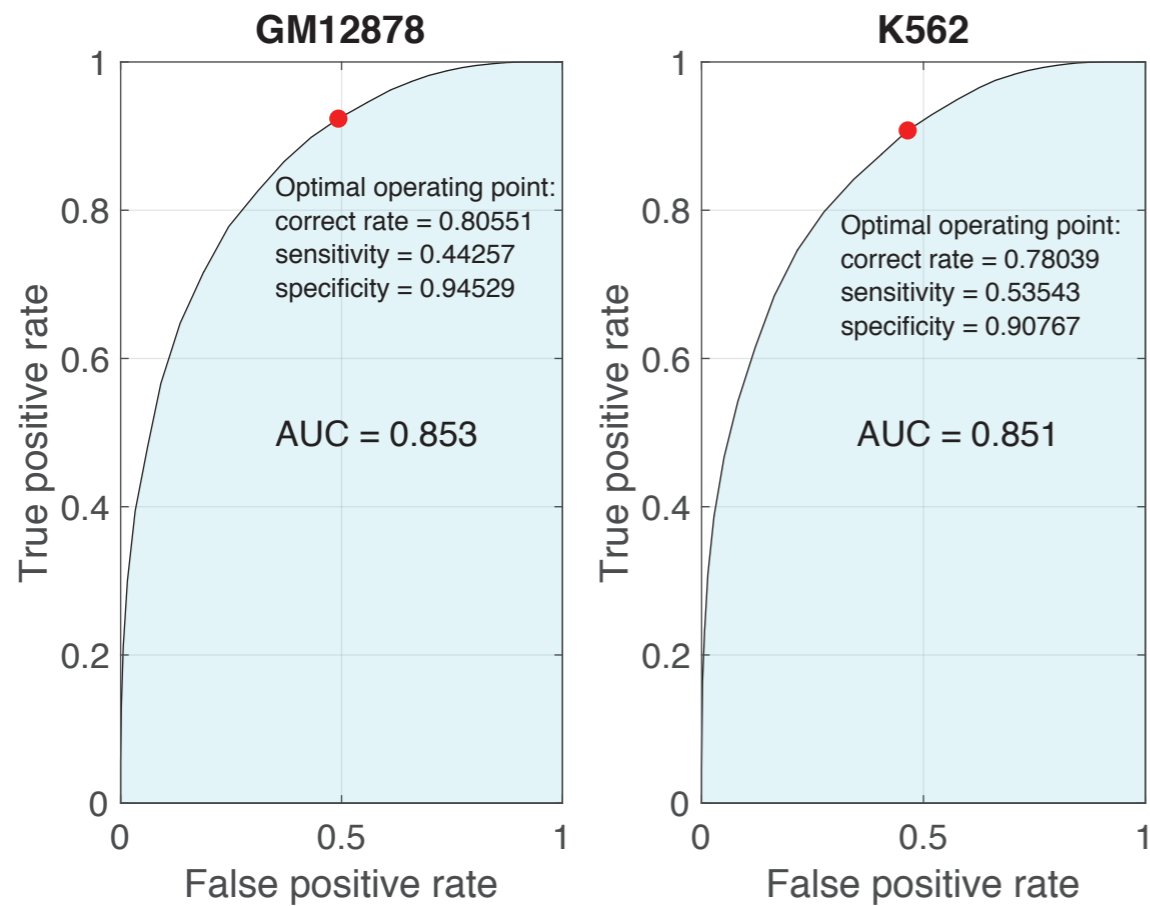

B

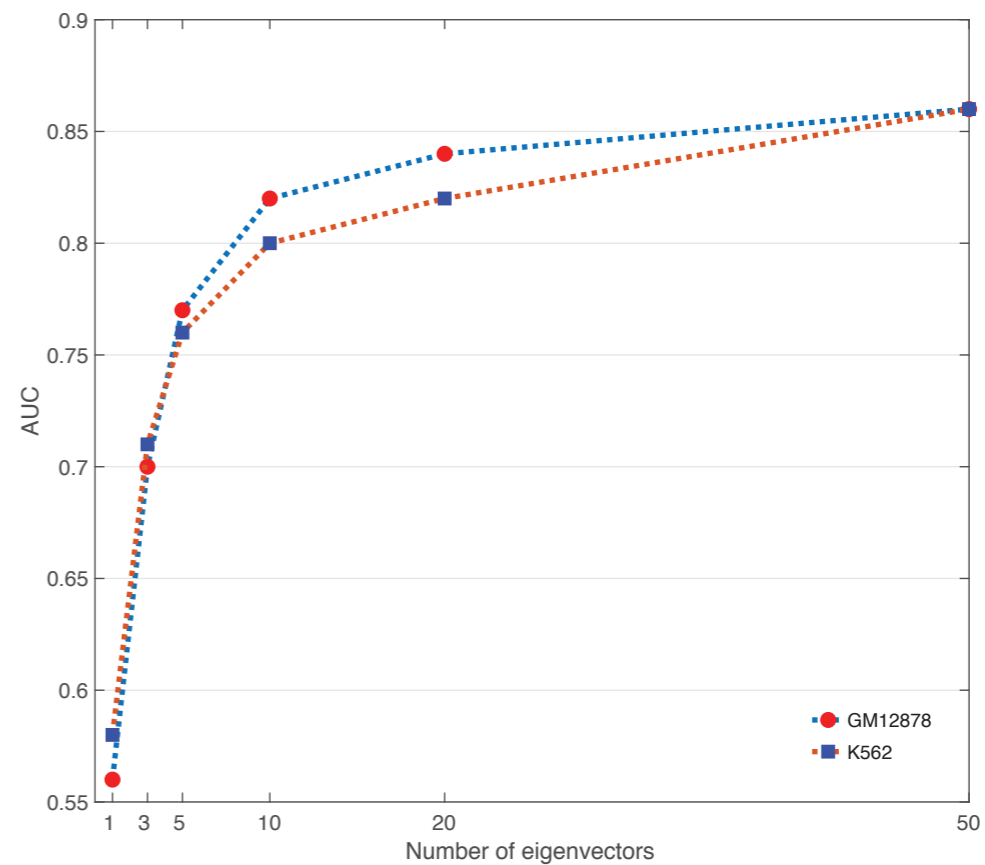

Supplement: Supplementary file 1 — Additional file 1: Figure S1. Pearson correlation coefficients between the gene co-expression matrix and three different matrices based on spatial positioning of genes: the CGP map (blue bars), the raw gene proximity map (green bars), and the normalized gene proximity map (yellow bars) for each of the 23 chromosomes for 10 ENCODE cell lines. Figure S2. (A) ROC curve for the gene compartment classification using leading eigenvectors of the CGP matrix for GM12878 and K562 cell lines. The horizontal axis is the false positive rate (1 − specificity) and the vertical axis is the true positive rate (sensitivity). The red dot indicates the optimal operating point. Components of the top 50 leading eigenvectors were used as features for the classification model. (B) Effect of the number of eigenvectors used in the gene compartment label classifier. The horizontal axis represents the number of eigenvectors in the CGP matrix used for model construction, ranged from 1 to 50. The vertical axis is the average AUROC of the resultant model over the 10-fold cross validation. The red circles and blue squares (almost completely coincide) represent the GM12878 and K562 cell lines respectively. Using the first leading eigenvector alone does not yield a good classification result. By additionally incorporating the second and third eigenvectors, the AUROC witnesses a dramatic increase (from 0.57 to 0.70). On the other hand, using more than 10 eigenvectors does not provide a substantial performance improvement any more. Figure S3. Objective function based on the empirical gene expression profile and randomized profiles, computed using the raw gene proximity map. The histogram for randomized profiles is normalized to have zero mean. A main difference between the plots generated from the CGP and the raw gene proximity map is that for cell lines RPMI-7951, SJCRH30 and SK-N-DZ, the value of the gene proximity map-based objective function generated from the empirical expression profile is mixed [file 12859_2020_3545_MOESM1_ESM.zip › FigureS2.pdf]

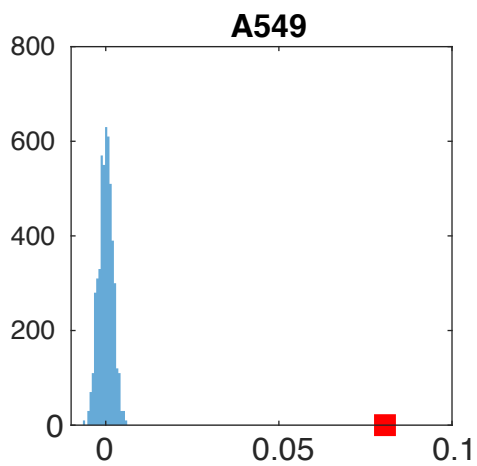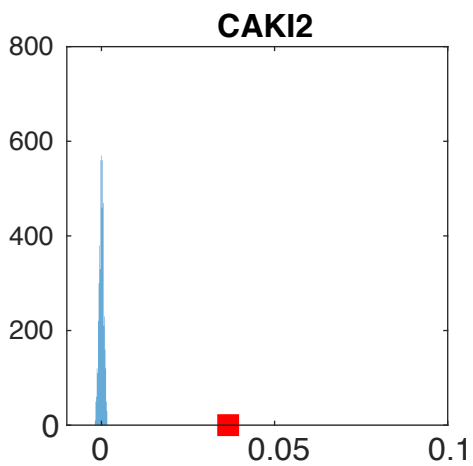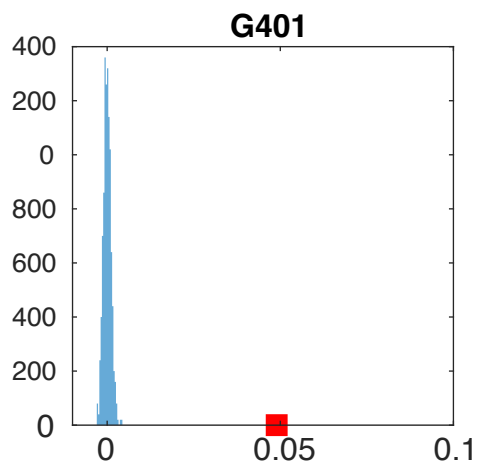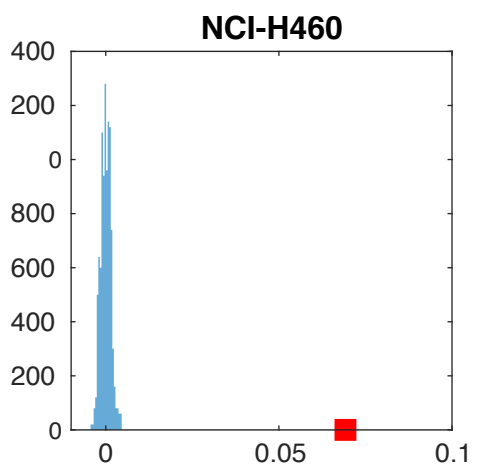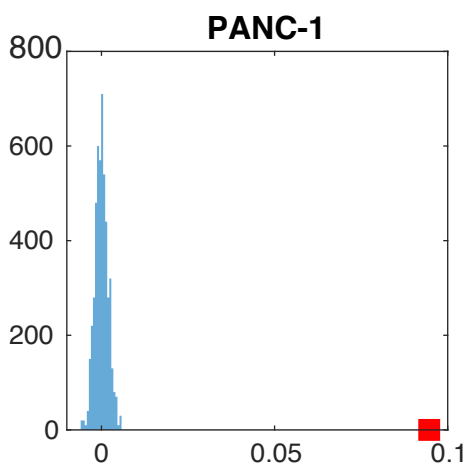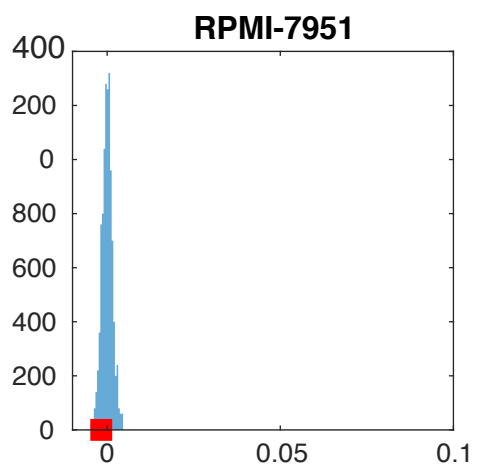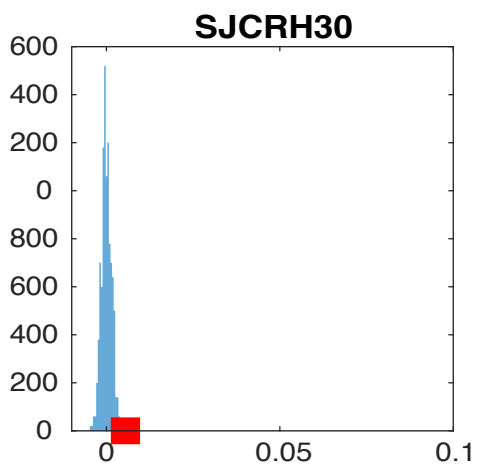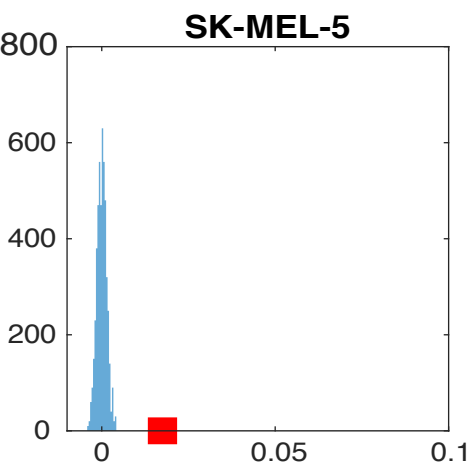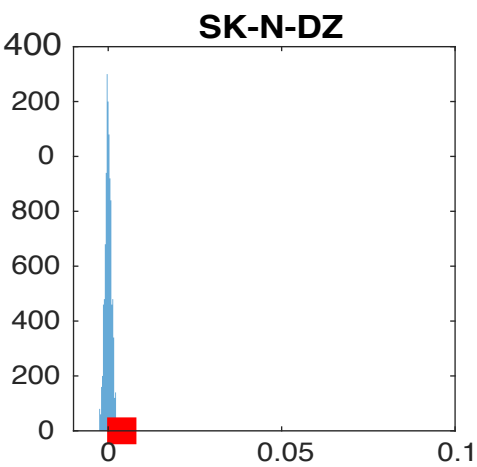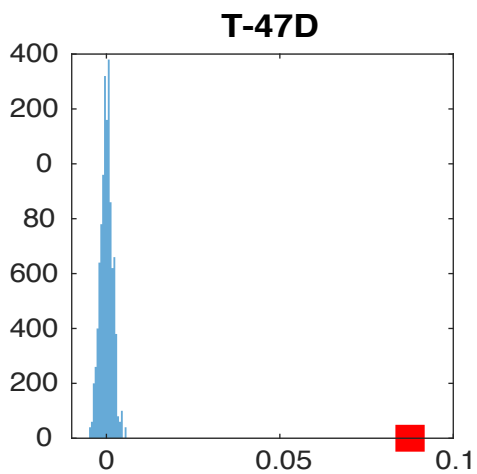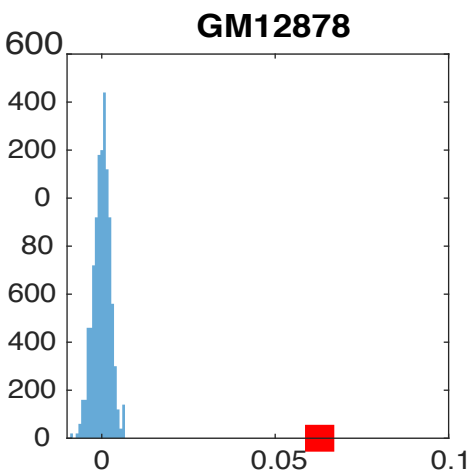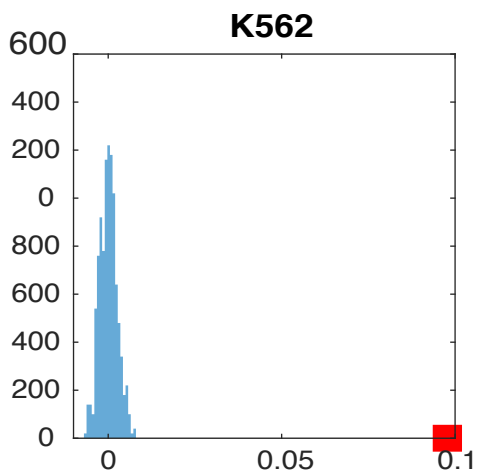

Supplement: Supplementary file 1 — Additional file 1: Figure S1. Pearson correlation coefficients between the gene co-expression matrix and three different matrices based on spatial positioning of genes: the CGP map (blue bars), the raw gene proximity map (green bars), and the normalized gene proximity map (yellow bars) for each of the 23 chromosomes for 10 ENCODE cell lines. Figure S2. (A) ROC curve for the gene compartment classification using leading eigenvectors of the CGP matrix for GM12878 and K562 cell lines. The horizontal axis is the false positive rate (1 − specificity) and the vertical axis is the true positive rate (sensitivity). The red dot indicates the optimal operating point. Components of the top 50 leading eigenvectors were used as features for the classification model. (B) Effect of the number of eigenvectors used in the gene compartment label classifier. The horizontal axis represents the number of eigenvectors in the CGP matrix used for model construction, ranged from 1 to 50. The vertical axis is the average AUROC of the resultant model over the 10-fold cross validation. The red circles and blue squares (almost completely coincide) represent the GM12878 and K562 cell lines respectively. Using the first leading eigenvector alone does not yield a good classification result. By additionally incorporating the second and third eigenvectors, the AUROC witnesses a dramatic increase (from 0.57 to 0.70). On the other hand, using more than 10 eigenvectors does not provide a substantial performance improvement any more. Figure S3. Objective function based on the empirical gene expression profile and randomized profiles, computed using the raw gene proximity map. The histogram for randomized profiles is normalized to have zero mean. A main difference between the plots generated from the CGP and the raw gene proximity map is that for cell lines RPMI-7951, SJCRH30 and SK-N-DZ, the value of the gene proximity map-based objective function generated from the empirical expression profile is mixed [file 12859_2020_3545_MOESM1_ESM.zip › FigureS3.pdf]

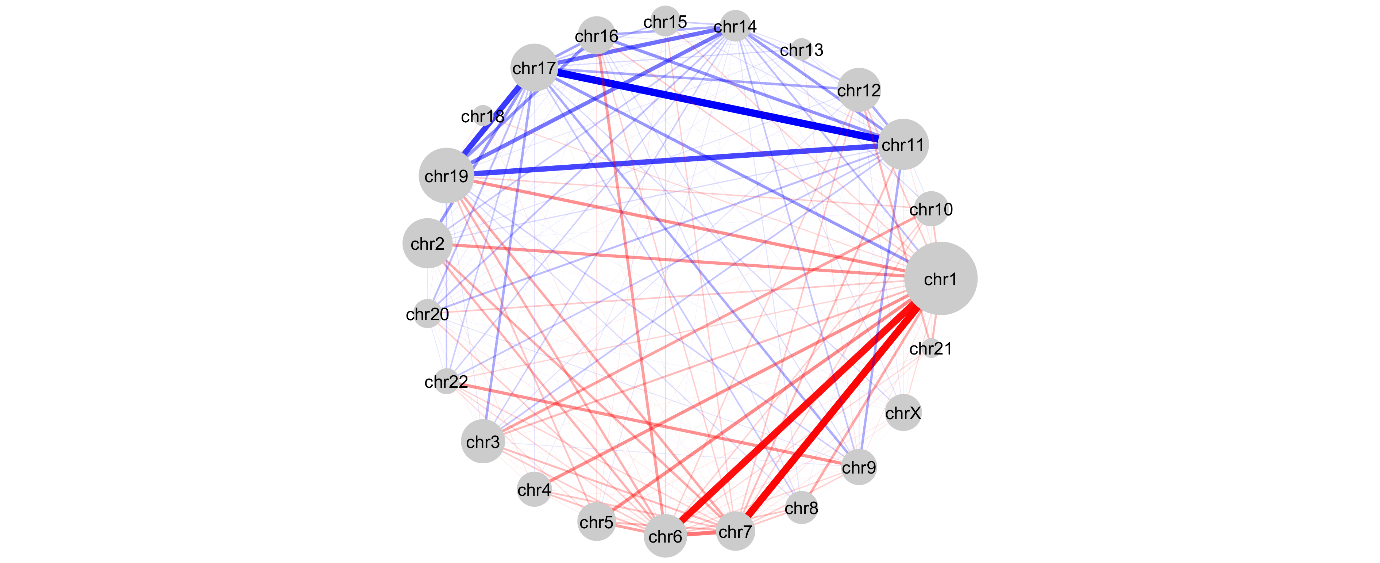

Supplement: Supplementary file 1 — Additional file 1: Figure S1. Pearson correlation coefficients between the gene co-expression matrix and three different matrices based on spatial positioning of genes: the CGP map (blue bars), the raw gene proximity map (green bars), and the normalized gene proximity map (yellow bars) for each of the 23 chromosomes for 10 ENCODE cell lines. Figure S2. (A) ROC curve for the gene compartment classification using leading eigenvectors of the CGP matrix for GM12878 and K562 cell lines. The horizontal axis is the false positive rate (1 − specificity) and the vertical axis is the true positive rate (sensitivity). The red dot indicates the optimal operating point. Components of the top 50 leading eigenvectors were used as features for the classification model. (B) Effect of the number of eigenvectors used in the gene compartment label classifier. The horizontal axis represents the number of eigenvectors in the CGP matrix used for model construction, ranged from 1 to 50. The vertical axis is the average AUROC of the resultant model over the 10-fold cross validation. The red circles and blue squares (almost completely coincide) represent the GM12878 and K562 cell lines respectively. Using the first leading eigenvector alone does not yield a good classification result. By additionally incorporating the second and third eigenvectors, the AUROC witnesses a dramatic increase (from 0.57 to 0.70). On the other hand, using more than 10 eigenvectors does not provide a substantial performance improvement any more. Figure S3. Objective function based on the empirical gene expression profile and randomized profiles, computed using the raw gene proximity map. The histogram for randomized profiles is normalized to have zero mean. A main difference between the plots generated from the CGP and the raw gene proximity map is that for cell lines RPMI-7951, SJCRH30 and SK-N-DZ, the value of the gene proximity map-based objective function generated from the empirical expression profile is mixed [file 12859_2020_3545_MOESM1_ESM.zip › FigureS4.png]
